# Supplementary material for: Complexome profiling of the Chlamydomonas psb28 mutant reveals TEF5 as an early PSII assembly factor
Source: Plant Cell. 2025 Mar 17;37(6):koaf055. doi: 10.1093/plcell/koaf055 (PMC12164590; doi:10.1093/plcell/koaf055)
Supplement: koaf055_Supplementary_Data [file koaf055_supplementary_data.zip › psb28_interactive.zip!/plots/intensity/qualityPlots/peptides/_Cre06.g278215.t1.1.html]

### Cre06.g278215.t1.1

Trivial name:   
SeqCoverage [median]: 11.40  
Mass: 43.31 kDa  
  
 

| Sequence | MissedCleavage | Mass [Da] | wtIntensity | muIntensity | Score |
| --- | --- | --- | --- | --- | --- |
| AADLSVVDNLIR | 0 | 1284.70 | 1527.56 | 0.00 | 81.34 |
| DAGFATHLVR | 0 | 1085.56 | 265.96 | 929.97 | 53.76 |
| GSVAVVTLNRPK | 1 | 1239.73 | 263.12 | 0.00 | 40.59 |
| IPELEAALVALGPR | 0 | 1447.84 | 528.61 | 0.00 | 74.94 |
|  |  |  | 2585.3 | 930.0 |  |
